# Prevalence of root fusion in canine maxillary second molar teeth using cone-beam computed tomography

**DOI:** 10.3389/fvets.2023.1306493

**Published:** 2023-11-30

**Authors:** Kristin Linder, Scott MacGee, Loren Schultz

**Affiliations:** ^1^Companion Animal Dentistry of Kansas City, Overland Park, KS, United States; ^2^Food Animal Production Medicine, University of Missouri, Columbia, MO, United States

**Keywords:** root fusion, radicular anatomy, maxillary second molar tooth, cone-beam computed tomography (CBCT), veterinary dentistry

## Abstract

This study evaluated the prevalence of root fusion in the right and left maxillary second molar teeth in dogs using cone-beam computed tomography (CBCT). A total of 180 dog CBCT scans, or a total of 360 maxillary second molar teeth, were analyzed in this study. Dogs were divided into weight categories of small (<10 kg), medium (10–25 kg), and large (>25 kg). Skull type (brachycephalic, mesocephalic, dolichocephalic) and sex were also recorded for each dog. Overall, 65% of maxillary second molar teeth had some type of root fusion. Of the teeth that had fusion, the only configuration represented was fusion of the distobuccal root with the palatal root. The most common root morphology overall (all dogs and both right and left maxillary second molar teeth included) was partial fusion (177/360, 49%). With the high prevalence of fused roots in maxillary second molar teeth in dogs found in this study, CBCT will help clinicians to more accurately assess a dog's anatomy and implications for treatment.

## 1 Introduction

Knowledge of radicular anatomy, with consideration to anatomical variations, is paramount to successful treatment outcomes. Fused roots pose a challenge from both an exodontic and endodontic perspective. As cone-beam computed tomography (CBCT) has become more widely used in veterinary dentistry, it has changed the way oral medicine is understood and practiced. CBCT has been shown to be superior to two-dimensional dental radiography in diagnostic yield for anatomic variations in abnormally shaped roots (1).

The maxillary second molar tooth in dogs is described as having three roots. To the author's knowledge, the prevalence of anatomic variation in the right and left maxillary second molar teeth of dogs has not been defined. However, anatomic variations in the dentition of the domestic cat have been described (2). The objective of this study is to describe and document the prevalence of root fusion in the right and left maxillary second molar teeth in dogs using CBCT.

## 2 Materials and methods

A total of 180 dog CBCT scans were randomly selected from the database of a dental specialty referral practice. The scans were randomly selected from dogs who required imaging for oral medicine, periodontic, endodontic, restorative, surgical, prosthodontic, or orthodontic procedures between January 2019 and May 2023. Dogs were positioned in sternal recumbency, and the tomographic images were viewed in sagittal, frontal, and transverse planes. Dogs missing the right and/or left maxillary second molar teeth, or dogs with mixed or deciduous dentition, were excluded from the study. The tomographic images were taken with a Planmed Verity device, with voxel size 200 μm, 6 mA, 88 kV. All tomographic studies were analyzed by one author (KL) using Planmeca Romexis software, version 5.3.4.39.

Dogs were divided into weight categories of small (<10 kg), medium (10–25 kg), and large (>25 kg); there were 60 dogs per weight group for a total of 180 dogs in the study. Skull type was assigned by breed (29 brachycephalic, 139 mesocephalic, 12 dolichocephalic). Sex (92 male, 88 female) was also recorded for each dog. To characterize the root morphology, the classification of Marcano-Caldera et al. was used with slight modification to account for veterinary patients (3):

- Separate: three separate roots existed at the level of the cementoenamel junction.
- Partial fusion: only two roots visualized at the level of the cementoenamel junction with evidence of periodontal ligament space between the three roots at any level apical to the furcation.
- Complete fusion: only two roots visualized at the level of the cementoenamel junction with no evidence of periodontal ligament space between the three roots at any level apical to the furcation.

A Chi-square test is used to interpret the relationship between two variables. Fisher's Exact tests also determine if there are associations between two variables, however, are utilized with smaller sample sizes. The Fisher's Exact test is used to test the overall model; if it is significant, then the Bonferroni adjustment is used for pairwise comparisons to find the significance. The data were analyzed using statistical software Stata SE 15.1. A *p* < 0.05 was considered significant.

## 3 Results

**Figure 1:**
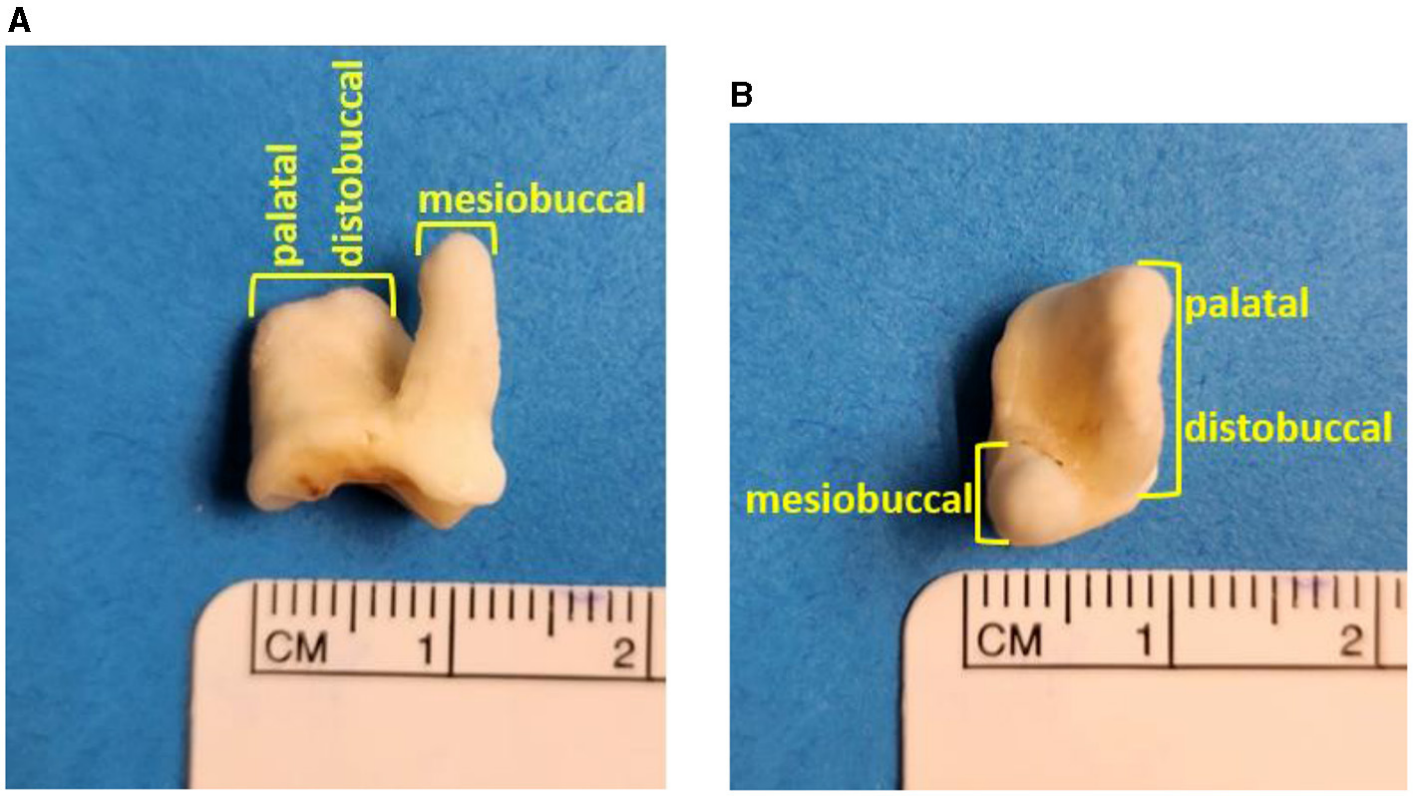
A left maxillary second molar tooth demonstrating complete fusion of the distobuccal and palatal roots: frontal view of left maxillary second molar tooth **(A)**, transverse view of left maxillary second molar tooth **(B)**.

**Figure 2:**
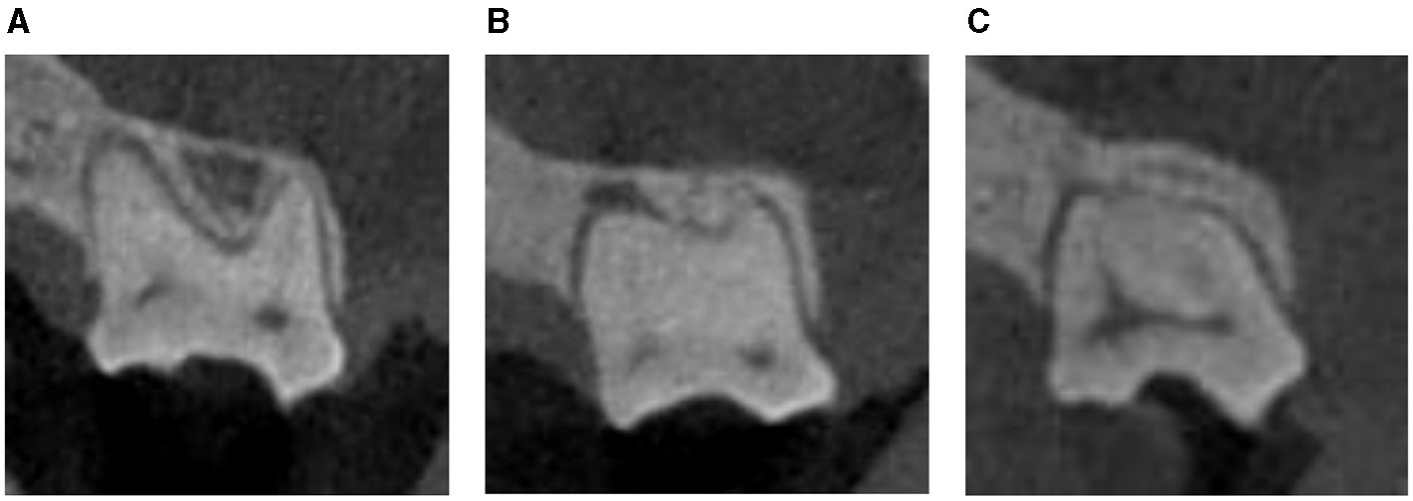
Examples of the different types of root morphology of the left maxillary second molar tooth as visualized on CBCT (frontal view): separate roots **(A)**, partial fusion **(B)**, complete fusion **(C)**.

A total of 180 dog CBCT scans, with a total of 360 maxillary second molar teeth, were analyzed in this study. Overall, 233/360 maxillary second molar teeth (65%) had some type of root fusion. Of the teeth that had some type of fusion, the only configuration represented was fusion of the distobuccal root with the palatal root (Figure 1). The variation in root morphology as visualized on CBCT imaging is illustrated in Figure 2.

**Figure 3:**
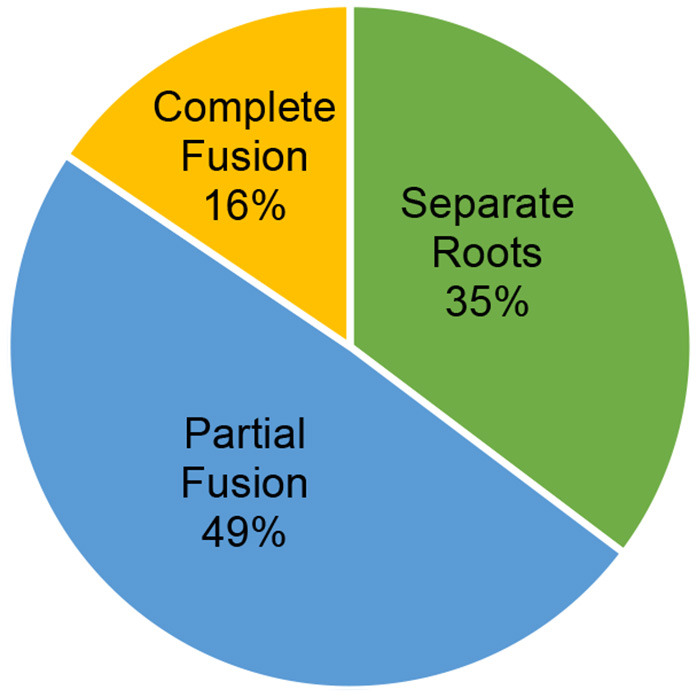
Prevalence of the varying root morphologies overall.

The most common root morphology overall (all dogs and both right and left maxillary second molar teeth included) was partial fusion (177/360, 49%), as illustrated in Figure 3.

**Figure 4:**
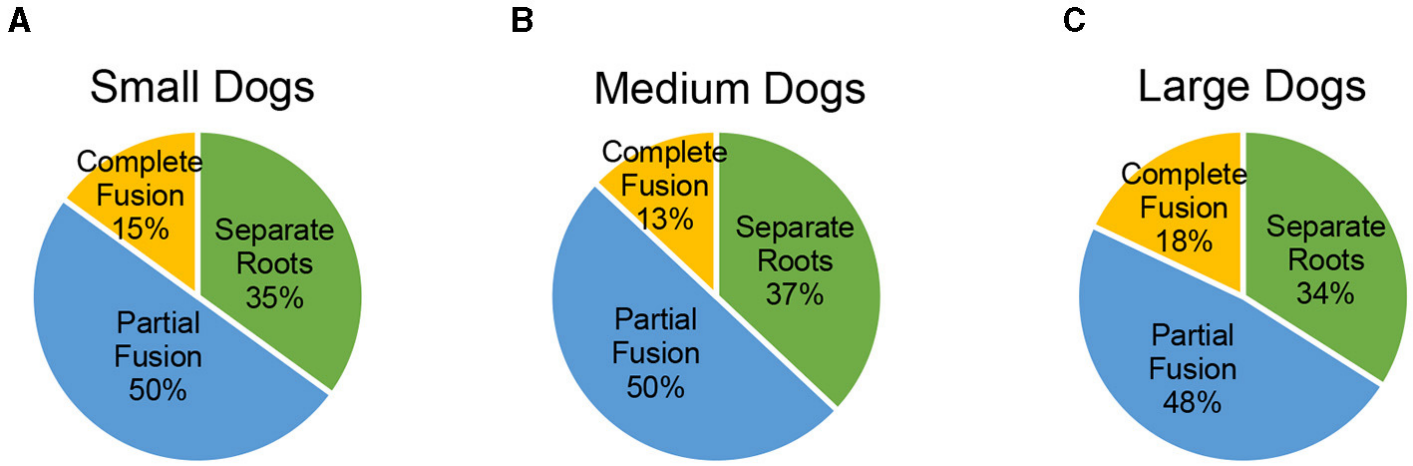
**(A–C)** Prevalence of the varying root morphologies in different weight categories.

The small weight group (60 dogs, 120 total maxillary second molar teeth) had 42/120 (35%) maxillary second molar teeth with separate roots, 60/120 (50%) with partial fusion, and 18/120 (15%) with complete fusion. The medium weight group (60 dogs, 120 total maxillary second molar teeth) had 44/120 (37%) maxillary second molar teeth with separate roots, 60/120 (50%) with partial fusion, and 16/120 (13%) with complete fusion. The large weight group (60 dogs, 120 total maxillary second molar teeth) had 41/120 (34%) maxillary second molar teeth with separate roots, 57/120 (48%) with partial fusion, and 22/120 (18%) with complete fusion. There was not a significant difference between weight groups and root morphology using a Chi-square analysis (*p* = 0.87613). The prevalence of the varying root morphologies in different weight categories is illustrated in Figure 4.

Within the entire population of dogs, 46/180 (26%) dogs had asymmetry: right maxillary second molar tooth root morphology did not match that of the left maxillary second molar tooth. Within the small weight category, there were 21/60 (35%) dogs with asymmetry. Within the medium weight category, there were 12/60 (20%) dogs with asymmetry. Within the large weight category, there were 13/60 (22%) dogs with asymmetry.

**Figure 5:**
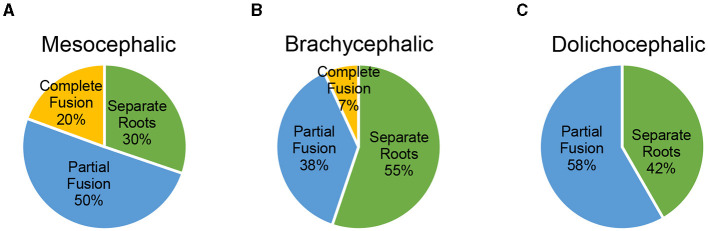
**(A–C)** Prevalence of the varying root morphologies among different skull types at the right maxillary second molar tooth.

Using a Fisher's Exact test to compare the difference in root morphology at the right maxillary second molar tooth, there was a statistically significant difference between skull types (*p* = 0.048). Since this was significant, a Bonferroni adjustment was used to investigate pairwise comparisons (mesocephalic vs. brachycephalic, mesocephalic vs. dolichocephalic, brachycephalic vs. dolichocephalic). The Bonferroni adjustment failed to find a difference in the pairwise comparisons at the right maxillary second molar tooth. The prevalence of the varying root morphologies among different skull types at the right maxillary second molar tooth is illustrated in Figure 5.

**Figure 6:**
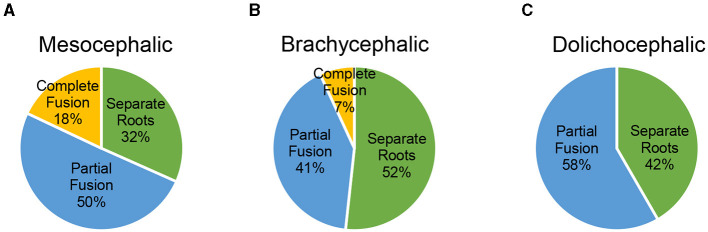
**(A–C)** Prevalence of the varying root morphologies among different skull types at the left maxillary second molar tooth.

Using a Fisher's Exact test to compare the difference in root morphology at the left maxillary second molar tooth, there was not a statistically significant difference between skull types (*p* = 0.119). The prevalence of the varying root morphologies among different skull types at the left maxillary second molar tooth is illustrated in Figure 6.

Using a Chi-square analysis, there was not a statistically significant difference between male and female root morphology at the right maxillary second molar tooth (*p* = 0.717), nor was there a statistically significant difference between male and female root morphology at the left maxillary second molar tooth (*p* = 0.277).

## 4 Discussion

Root formation begins following crown development in the bell stage of odontogenesis (4). The cervical loop forms at the junction of the outer enamel epithelium and the inner enamel epithelium (4). The cervical loop cells proliferate to form a two-layer structure known as Hertwig's Epithelial Root Sheath (HERS), which guides root formation (4). HERS continues to grow by rapid mitotic division and eventually angles back toward the center, forming the epithelial diaphragm (4). If there are errors involving the epithelial diaphragm, alterations in root number, length, and shape may occur (4). A human study on tooth root malformations demonstrated that the apical growth of HERS associated with root elongation and furcation formation in multirooted teeth appeared rather susceptible to adverse effects, resulting in frequent anatomical radicular variation (5).

The current study establishes a high prevalence of root fusion in maxillary second molar teeth in dogs; nearly two-thirds (65%) of the dogs in the study had some degree of root fusion. Root fusion and difficulty visually isolating roots due to conformation may introduce complications during exodontic procedures. Advanced knowledge from a CBCT study demonstrating variations in radicular anatomy is helpful when sectioning a tooth for exodontia, as well as in surgical planning. Teeth with separate roots may need to be sectioned prior to extraction, while teeth with completely fused roots negate the need for sectioning the fused roots. Additionally, there are implications with root fusion in endodontic therapy. Although root fusion can have implications in endodontic therapy, it is unlikely that endodontic therapy would be performed in a maxillary second molar tooth in veterinary dentistry. In humans, the presence of root fusion has been associated with complex root canal systems; prior knowledge of morphologic variation is paramount to the success of endodontic therapy (6).

In multi-rooted teeth with superimposition of roots, variations in root morphology can be difficult to interpret on two-dimensional dental radiographs. In humans, three-dimensional analysis with CBCT was shown to provide superior diagnostic imaging detection for root abnormalities such as cementum union in concrescence (7). Additionally, in a recent study of small to medium-sized brachycephalic dogs, the diagnostic yield for CBCT was found to be significantly higher than dental radiography in four categories, including abnormally shaped roots. In fact, maxillary second molar teeth with abnormally shaped roots were identified only with serial CBCT slices and custom cross sections (8).

Future studies with a more even distribution of skull types (larger numbers of brachycephalic and dolichocephalic) are necessary to determine the significance, if any, between root morphology and skull shape. While there was a significant difference in the overall comparison of skull types at the right maxillary second molar tooth, the Bonferroni adjustment failed to find this difference. This is likely due to the low number of dolichocephalic skulls, therefore lacking enough power to find a difference.

With the high prevalence of fused roots in maxillary second molar teeth in dogs found in this study, CBCT will help clinicians to more accurately assess a patient's anatomy and implications for treatment, resulting in shorter extraction times and reduced morbidity.

## Data availability statement

The raw data supporting the conclusions of this article will be made available by the authors, without undue reservation.

## Author contributions

KL: Writing—original draft. SM: Writing—original draft. LS: Writing—original draft.
